# Laparoscopic splenectomy using conventional instruments

**DOI:** 10.4103/0972-9941.19000

**Published:** 2005-09

**Authors:** H. G. Doctor

**Affiliations:** Consultant Surgeon, Jain Clinic Hospital and Bhatia General Hospital, Mumbai, India, E-mail: hdoctor@jnin.jnj.com

Dear Sir,

I congratulate Dr Dalvi and others for their original article on ‘Laparoscopic splenectomy using conventional instruments.’[1] It is highly commendable that they have sucessfully performed 26 splenectomies using reusable conventional instruments, monopolar and bipolar cautery, clips and intracorporeal knotting. There was no mortality and minimal morbidity during a follow up of 6 years. It was interesting to read about their annovation of ‘glove-in-glove’ used as a hand port. I wish they had provided a diagram to indicate the position of the patient and port-sites. This also shows what can be achieved by commitment and dedication of a surgical team in a teaching medical college and hospital when even today many teaching colleges have yet to take off in minimal access surgery.
